# Medical Association of Georgia

**Published:** 1868

**Authors:** 


### MEDICAL ASSOCIATION OF GEORGIA.

The next meeting of this body will be held on the second Wednesday (8th day) of April, and Augusta was selected as the place for the session of the present year. As evidence of the interest manifested by members present at the meeting in Griffin last year, we would mention the fact that committees were appointed to revise the constitution and bylaws, to receive and examine prize Essays, etc., etc. From notices in the last issue of our Journal it seems that some, as least, of the committees appointed are alive to the duties assigned them. By all means let us have a correct and full revision of the constitution and by-laws. Those now of force are in detached portions, passed by the body at different times, and many of them published only in the various Medical journals, copying the transactions. In order to know what they are as a whole, compilation and revision are altogether necessary, and it is hoped the committee will present at the meeting in Augusta, a code such as may be authorized by the various changes and additions made from time to time. Nothing is more conducive to the uprightness, advancement and usefulness of the profession, than the annual meetings of physicians in the State, properly conducted. Every respectable graduate in Medicine can be a member by making application at a meeting of the Association, and as the place of meeting is changed every year, from place to place, most physicians have an opportunity of doing so at some time or other, without inconvenience. Though the stringency in pecuniary matters will prevent the attendance of many in distant portions of the State, at the approaching meeting in Augusta, yet we hope to have a reunion of the brotherhood, particularly from Eastern Georgia, which will inspire members with enthusiasm in scientific investigations for the next twelve months.

The committee of arrangement will doubtless secure, if possible, the passage of members at half fare, as usual, over the several railroads.

